# The Hrk1 kinase is a determinant of acetic acid tolerance in yeast by modulating H^+^ and K^+^ homeostasis

**DOI:** 10.15698/mic2023.12.809

**Published:** 2023-11-14

**Authors:** Miguel Antunes, Deepika Kale, Hana Sychrová, Isabel Sá-Correia

**Affiliations:** 1 iBB—Institute for Bioengineering and Biosciences, Instituto Superior Técnico, Universidade de Lisboa, 1049-001 Lisbon, Portugal.; 2 Department of Bioengineering, Instituto Superior Técnico, Universidade de Lisboa, 1049-001 Lisbon, Portugal.; 3 Associate Laboratory i4HB—Institute for Health and Bioeconomy at Instituto Superior Técnico, Universidade de Lisboa, Av. Rovisco Pais, 1049-001 Lisbon, Portugal.; 4 Laboratory of Membrane Transport, Institute of Physiology, Czech Academy of Sciences, Videnska 1083, 142 00 Prague 4, Czech Republic.

**Keywords:** acetic acid tolerance, Pma1 activity, plasma membrane H+-ATPase, Nha1, Saccharomyces cerevisiae, yeast kinases, NPR/Hal family

## Abstract

Acetic acid-induced stress is a common challenge in natural environments and industrial bioprocesses, significantly affecting the growth and metabolic performance of *Saccharomyces cerevisiae*. The adaptive response and tolerance to this stress involves the activation of a complex network of molecular pathways. This study aims to delve deeper into these mechanisms in *S. cerevisiae*, particularly focusing on the role of the Hrk1 kinase. Hrk1 is a key determinant of acetic acid tolerance, belonging to the NPR/Hal family, whose members are implicated in the modulation of the activity of plasma membrane transporters that orchestrate nutrient uptake and ion homeostasis. The influence of Hrk1 on *S. cerevisiae* adaptation to acetic acid-induced stress was explored by employing a physiological approach based on previous phosphoproteomics analyses. The results from this study reflect the multifunctional roles of Hrk1 in maintaining proton and potassium homeostasis during different phases of acetic acid-stressed cultivation. Hrk1 is shown to play a role in the activation of plasma membrane H^+^-ATPase, maintaining pH homeostasis, and in the modulation of plasma membrane potential under acetic acid stressed cultivation. Potassium (K^+^) supplementation of the growth medium, particularly when provided at limiting concentrations, led to a notable improvement in acetic acid stress tolerance of the *hrk1*Δ strain. Moreover, abrogation of this kinase expression is shown to confer a physiological advantage to growth under K^+ ^ limitation also in the absence of acetic acid stress. The involvement of the alkali metal cation/H^+ ^ exchanger Nha1, another proposed molecular target of Hrk1, in improving yeast growth under K^+ ^ limitation or acetic acid stress, is proposed.

## INTRODUCTION

The yeast *Saccharomyces cerevisiae* must constantly adapt to changing and challenging environments, both in nature and industrial bioprocesses, to thrive and maintain cellular homeostasis under a wide range of stresses [1–4]. To achieve this, a complex network of molecular pathways is coordinately activated, controlling stress response and cell growth in *S. cerevisiae* [5–7]. Of particular significance among these challenges is the adaptation to acetic acid stress, a central focus of this work. Acetic acid is present in lignocellulosic biomass hydrolysates and is one of the main growth and metabolism inhibitors associated with this sustainable feedstock for the production of biofuels and bulk chemicals by second-generation (2G) integrated biorefinery processes [8]. Acetic acid is also an inhibitory metabolite of yeast fermentation and a commonly used food preservative [4, 9].

The adaptive response to acetic acid stress in yeast involves the interplay of diverse cellular and metabolic processes [4, 10, 11]. One of the main effectors is the transcription factor Haa1, which is responsible for the direct or indirect transcriptional activation of 80% of acetic acid-responsive genes, including the gene encoding the Hrk1 kinase [12–14]. Hrk1 is an important determinant of acetic acid tolerance and a member of the NPR/Hal family of kinases [13, 15]. These yeast-specific kinases have been implicated in the modulation of fundamental biological processes, primarily through the regulation of plasma membrane proteins involved in nutrient uptake and metabolism, ion homeostasis, and tolerance to environmental stresses (reviewed in [15]). Two phosphoproteomics analyses have suggested several potential targets for the activity of the Hrk1 protein kinase [16, 17]. A membrane-associated quantitative phosphoproteomics study performed in our lab revealed that Hrk1 may be involved in modulating the phosphorylation levels of several membrane proteins under non-stressing conditions and during early response to acetic acid-induced stress [16]. Among these phosphoproteins, multidrug resistance (MDR) transporters of the major facilitator superfamily (MFS) Tpo3, Tpo4, and Qdr2 [18–20], as well as plasma membrane H^+^-ATPase Pma1, exhibited more marked Hrk1-dependent alterations in phosphorylation levels in response to acetic acid stress than under non-stressing conditions [16]. The other quantitative systems-level phosphoproteomics study, conducted under non-stressing growth conditions, confirmed several protein targets and uncovered additional potential targets for Hrk1. These proteins include the Na^+^,K^+^/H^+^ antiporter Nha1, the MDR transporter Tpo4, and the ATP-binding cassette (ABC) multidrug transporter Yor1 [17].

The gene *PMA1* encodes the major form of yeast plasma membrane H^+^-ATPase being the first biological target attributed to Hrk1 [21]. Although Hrk1 was reported to be involved in the mild glucose-activation of this proton pump, the impact of Ptk2, a kinase belonging to the same family as Hrk1, is substantially higher [15, 21]. Pma1 is an essential enzyme for yeast physiology, as a null mutation in haploid cells is lethal [22].

Its main function is actively pumping protons outside the cells, creating an electrochemical gradient that allows nutrient uptake through secondary transporters [23]. Moreover, Pma1 contributes to the control of intracellular pH, maintaining it between 6.0 and 7.0 even amidst large fluctuations in extracellular pH [24]. Notably, the activity of this H^+^ pump is the major ATP consumer in the cell, accounting for up to 20% of the cellular ATP utilized in actively growing cells under glucose metabolism [25]. Given its substantial role, the proper modulation of this proton pump is indispensable for optimal cellular functioning. Under acetic acid or other weak acid-induced stress, Pma1 emerges as a key determinant of tolerance, as demonstrated by the enhanced tolerance to acetic acid in *PMA1*-overexpressing strains [26–28]. This observation is also consistent with the activation of plasma membrane H^+^-ATPase activity in cells grown in acid media [29], where the increased activity rather than increased levels of the enzyme, has been identified as the main activation cause [30]. Such activation has also been registered under weak acid stress [31–34]. Another relevant potential Hrk1-target transporter is the Na^+^,K^+^/H^+^ antiporter Nha1, a secondary active transport system that utilizes the inward gradient of protons created by the Pma1 H^+^-ATPase [35]. Nha1 serves a dual role in yeast cells, as it eliminates surplus K^+^ and detoxifies cells by removing other toxic alkali metal cations, such as Na^+^ and Li^+^ [36, 37]. This potassium transporter, as well as the K^+^ importers Trk1/Trk2 [38], have been shown to impact plasma membrane potential, intracellular pH [29–44] and tolerance to weak acid stress [41, 45–47]. Potassium availability was found to be essential for the achieving of maximum tolerance to acetic acid [46, 47] and formic acid [48], even though the expression of *TRK1* has opposing effects in yeast tolerance to these weak acids [48]. The level of tolerance increases in tandem with the rise in K^+^ concentration in the growth medium until a saturating concentration is reached [46–48]. The concentrations of monovalent ions, such as H^+^, K^+^ and Na^+^, determine many physiological parameters such as cell volume, plasma membrane potential and intracellular pH [36, 49]. In yeast cells, these parameters are maintained within a narrow range during the adaptation to external perturbations, including ionic, osmotic and alkaline pH stress [50–52]. Proper regulation of ion homeostasis is a crucial process for the survival and proper functioning of yeast cells in the presence or absence of environmental stress [49, 53,]. This involves the regulation of several ion transporters and channels, modulating ion concentrations in the extracellular and intracellular environments, which can be seriously disrupted under stress [49, 54–56]. The alkali-metal-cation transporters mediate active influx and efflux of K^+^, contributing significantly to potassium and proton homeostasis [38, 57–59]. Together, the coordinated activity of Pma1 and the alkali-metal-cation transporters ensures the delicate balance of intracellular potassium and proton levels [36, 54].

This study aimed to investigate further acetic acid stress tolerance mechanisms in *S. cerevisiae* focusing on the role of the Hrk1 kinase by exploring a physiological approach suggested by previous phosphoproteomics analyses. Studies examined the involvement of Hrk1 in modulating plasma membrane H^+^-ATPase activity, the intracellular pH (pHi), and plasma membrane potential during the cultivation of a population of acetic acid-stressed and non-stressed cells. Additionally, the cross-talks between Hrk1, K^+^ homeostasis and acetic acid tolerance were examined.

## RESULTS

### *HRK1* expression allows maximal activation of plasma membrane H^+^-ATPase activity during acetic acid-stress cultivation

**Figure 1:**
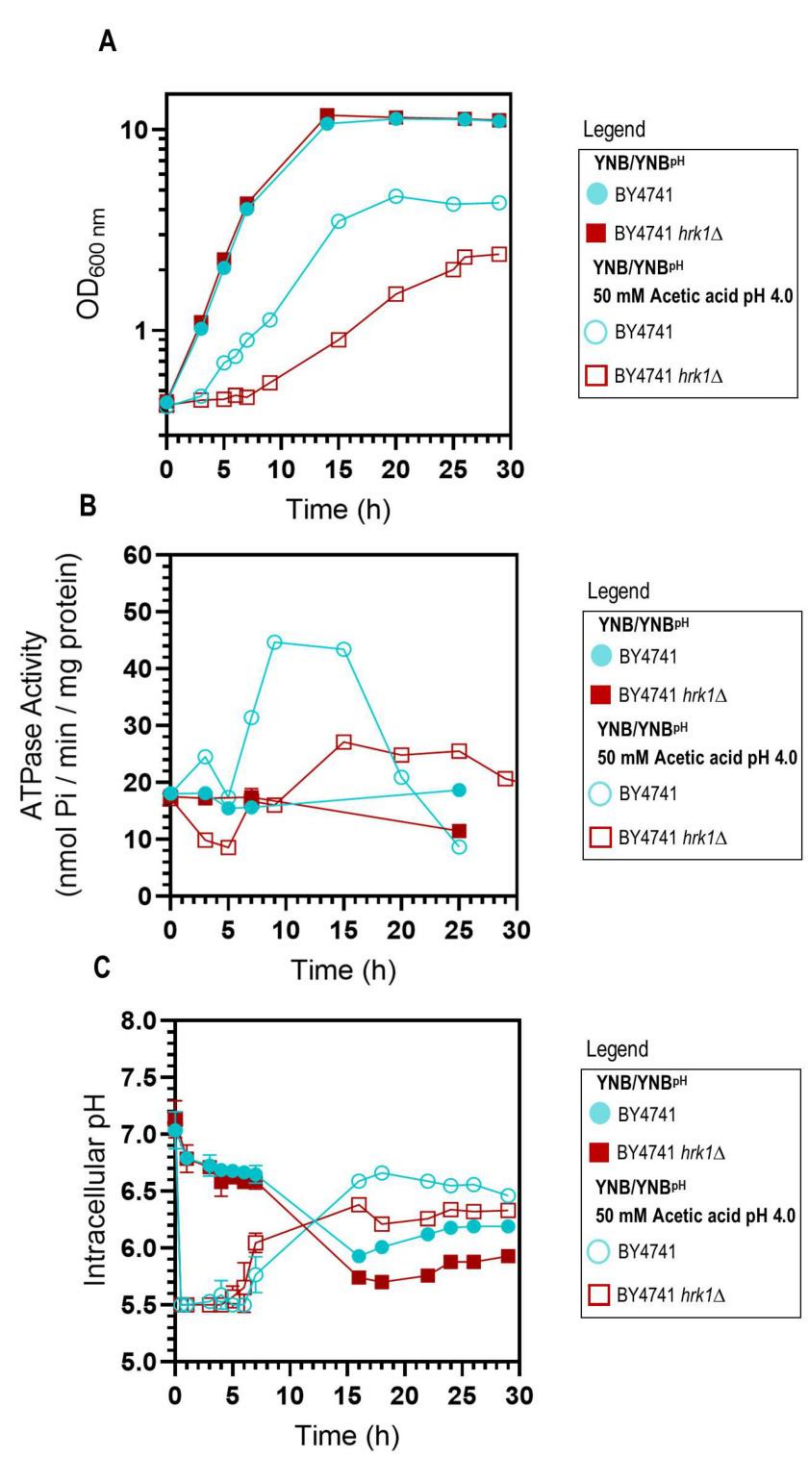
FIGURE 1: Comparative analysis of physiological parameters of the parental and *hrk1*Δ strains under acetic acid-stressed and non-stressed cultivations. Growth curves **(A)**, plasma membrane H^+^-ATPase activity **(B)**, and intracellular pH (pHi) **(C)** of *S. cerevisiae* BY4741 (parental strain ●/○) and the *hrk1*Δ deletion mutant strain (■/□) cultivated in YNB/YNB^pH^ medium (●/■) or in YNB/YNB^pH^ supplemented with 50 mM acetic acid (○/□) at pH 4.0. The displayed results for ATPase activity measurements are derived from an experiment representative of other independent experiments leading to similar results. In the case of pHi, the data presented represents average values ± SD of at least two independent experiments.

As previously described [47], the deletion of the *HRK1* gene in *S. cerevisiae* BY4741 leads to heightened sensitivity to acetic acid stress. This is characterized by a prolonged phase of growth latency, decreased maximum specific growth rate, and reduced final biomass production (**Figure 1A**), underscoring the critical role played by Hrk1 in orchestrating the cellular adaptive response to acetic acid-induced stress. To investigate the hypothesized impact of Hrk1 on the activation of Pma1 under acetic acid stress, the plasma membrane H^+^-ATPase activity in purified cell membranes was assessed (**Figure 1B**). Pma1 activity is crucial in the response to acetic acid stress and is a putative phosphorylation target of Hrk1 [16]. Results reveal a significant increase of H^+^-ATPase activity in cells of the parental strain during growth under acetic acid stress (up to 2.4-fold, compared with unstressed cells; **Figure 1B**). A substantial reduction of more than one-third in the maximal activation of Pma1 activity was registered in the *hrk1*Δ mutant under acetic acid stress conditions compared to the parental strain (**Figure 1B**). However, the time-course profile of proton pump activation during the growth curves of acetic acid-stressed parental and *hrk1*Δ cells are similar. Activation occurred as the cell culture transitioned from the growth latency phase to the exponential phase of acetic-adapted growth. The differences in growth efficiency under acetic acid stress between these two cultures correlate with variations in the activation profiles of this essential membrane-active transporter. These results emphasize the major role of Hrk1 kinase in facilitating the activation of the key tolerance determinant Pma1 in response to acetic acid-induced stress. However, no significant differences were observed in the basal activity of glucose-grown cells under non-stressing conditions.

### *HRK1* expression plays a role in maintaining pH homeostasis during acetic acid-stressed cultivation

Considering that Pma1 is regarded as the primary regulator of yeast cytosolic pH and considering the differences observed in the Pma1 activation profile of acetic acid-stressed and unstressed cultures, expressing or not the *HRK1* gene, the pHi of both the *hrk1*Δ deletion mutant and parental strains was estimated using the ratiometric probe pHluorin. Exposure to acetic acid caused an initial rapid and pronounced decrease in pHi, dropping from approximately pH 7.0 to 5.5 in the parental and *hrk1*Δ strains (Figure 1C). Considering that the pHi determination method employed is limited by the sensitivity range of pHluorin (from 5.5 to 8.0), for the concentration of acetic acid used (50 mM pH 4.0) it was not possible to rigorously evaluate the lowest pHi reached by both the parental and *hrk1*Δ strains. However, it is clear that the *hrk1*Δ strain displayed a diminished ability to restore pHi to more physiological values, achieving lower maximal pHi values compared with the parental strain (**Figure 1C**). The time-course of the pHi profiles for the parental and the *hrk1*Δ cultures was consistent with the time-course described for the activity values of the plasma membrane H^+^-ATPase for both strains (**Figure 1B** and **C**). Results support the concept that *HRK1* expression plays a role in maintaining pH homeostasis under acetic acid stress, strongly suggesting that the mechanisms underlying Hrk1 activity as an acetic acid tolerance determinant are intertwined with its function in preserving pHi balance.

### *HRK1* expression has an impact on plasma membrane electrochemical potential during acetic acid-stressed cultivation

**Figure 2:**
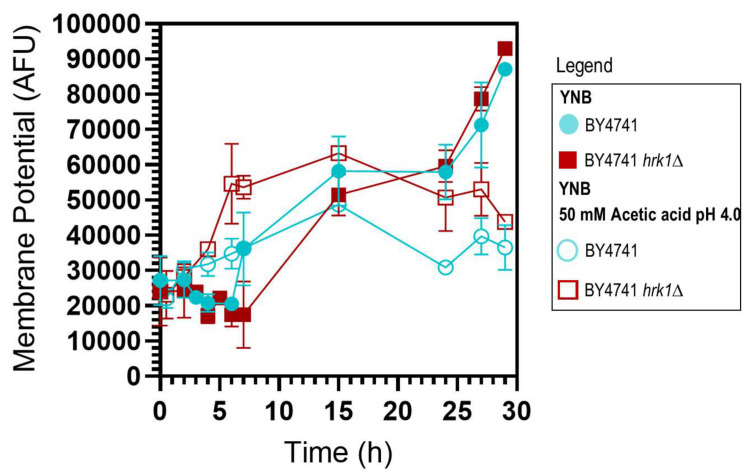
FIGURE 2: Estimation of plasma membrane potential of *S. cerevisiae* BY4741 and BY4741 *hrk1*Δ under acetic acid-stressed and non-stressed cultivations. Plasma membrane potential (Arbitrary fluorescence units) of BY4741 (●/○) and BY4741 *hrk1*Δ (■/□) cultivated in YNB medium pH 4.0 in the presence (○/□) or absence (●/■) of acetic acid stress (50 mM acetic acid at pH 4.0). The data presented represents average values ± SD of at least two independent experiments.

The establishment of an electrochemical gradient across the plasma membrane governs the uptake of essential nutrients and ions required for proper cellular function and the expulsion of toxic metabolites and other toxicants. The estimation of plasma membrane potential was conducted along the cultivation of BY4741 and BY4741 *hrk1*Δ cultures, in the presence or absence of acetic acid stress. Over the course of exponential growth in both unstressed cell cultures, a depolarization of the plasma membrane was observed, which was gradually restored once cells reached mid-exponential phase (**Figure 2**). Upon sudden exposure to acetic acid, a slight initial depolarization during the lag phase was registered in both cultures. The parental strain was able to progressively restore the membrane potential throughout the exponential growth phase, albeit with an increase in membrane hyperpolarization that was consistently lower than that observed in unstressed cells (**Figure 2**). Conversely, exposure of *hrk1*Δ cells to acetic acid resulted in plasma membrane hyperpolarization that continued to intensify throughout the lag phase of growth and stabilized to the maximal values of unstressed cells once exponential growth was initiated (**Figure 2**).

### *HRK1* and *NHA1* expression have an effect in improving yeast growth under K^+ ^ limitation in the absence or presence of acetic acid stress

**Figure 3:**
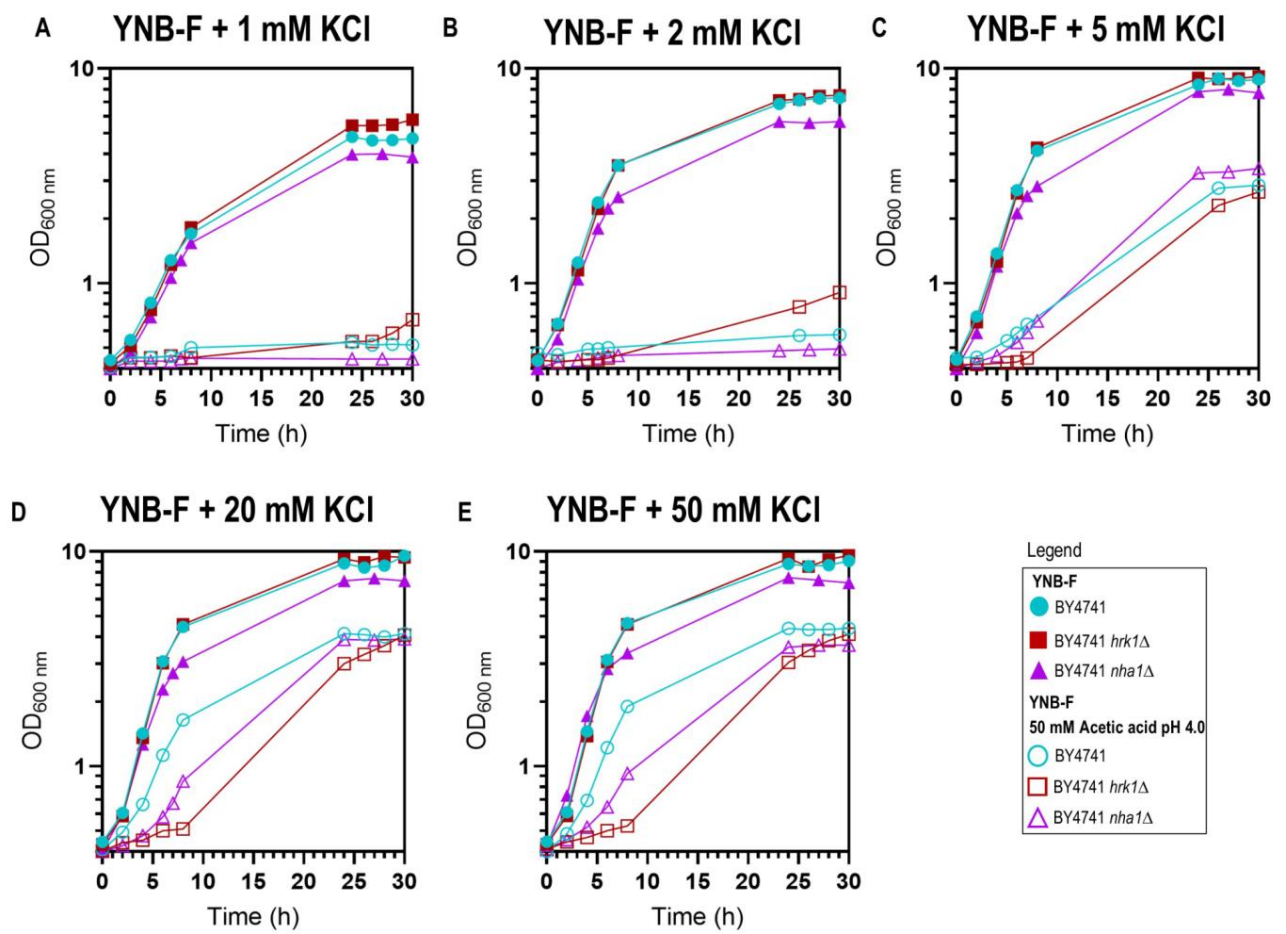
FIGURE 3: Effect of potassium (K^+^) supplementation in yeast growth in YNB-F medium pH 4.0 supplemented or not with 50 mM acetic acid. Growth curves of the parental (●/○), *hrk1*Δ (■/□), and *nha1*Δ (▲/▵) strains in the absence (●/■/▲) or presence (○/□/▵) of acetic acid stress in media supplemented with various concentrations of KCl – 1 mM **(A)**, 2 mM **(B)**, 5 mM **(C)**, 20 mM **(D)**, and 50 mM **(E)**. The results are shown as a mean of two independent experiments.

Prior studies have demonstrated that potassium (K^+^) supplementation results in the improvement of growth under acetic acid [46, 47] and formic acid [48] stresses, with the extent of improvement dependent on the potassium concentration until a saturating concentration is reached. In the present study, this phenomenon was evident in both the parental and the *hrk1*Δ strains since, below 20-50 mM KCl concentrations in the medium, K^+^ supplementation led to the reduction in the duration growth latencies allowing growth in the presence of the tested acetic acid concentration (50 mM at pH 4.0; **Figure 3**). Remarkably, for limiting potassium concentrations (1 mM and 2 mM KCl added to YNB-F) and under acetic acid stress conditions, the *hrk1*Δ culture displayed shorter lag phases compared to the parental strain. Moreover, this mutant was able to initiate exponential growth at limiting K^+^ concentrations (1 mM and 2 mM), while, after 30 h of incubation, the parental strain did not (**Figure 3A**). In the absence of stress, under K^+^ limitation (1 mM KCl added to YNB-F), the *hrk1*Δ deletion mutant strain also appears to exhibit superior growth compared to the parental strain, reaching a higher final biomass concentration (**Figure 3A** and **B**). This indicates that the absence of *HRK1* confers an advantage in terms of growth under conditions of limited potassium availability. These results led us to postulate the involvement of Hrk1 in the modulation of K^+^ homeostasis. Making use of a former phosphoproteomics study [17] to retrieve potential Hrk1 phosphorylation targets, the K^+^,Na^+^/H^+^ antiporter Nha1 emerged as a promising biological target for further investigation.

To investigate the possible role of Hrk1 in the modulation of potassium homeostasis through Nha1, particularly under acetic acid stress, the growth of the strain lacking the Nha1 transporter was also monitored under various potassium supplementation concentrations and in the presence or absence of acetic acid stress (**Figure 3**). Throughout the range of tested potassium concentrations (1, 2, 5, 20, and 50 mM), the *nha1*Δ mutant strain consistently displayed a slightly slower growth rate and a notably reduced final biomass concentration, in contrast to the parental and *hrk1*Δ strains (**Figure 3**). Deletion of the *NHA1* gene resulted in increased sensitivity to acetic acid stress characterized by the presence of a longer lag phase than the parental strain in the presence of saturating potassium concentrations (**Figures 3D** and **E**). Furthermore, increasing potassium supplementation concentrations also led to increasingly improved *nha1*Δ growth under acetic acid stress. Under conditions of limiting potassium concentrations (1 mM or 2 mM K^+^ added to YNB-F), and under acetic acid stress, the *nha1*Δ could not initiate exponential growth similarly to the parental strain (**Figures 3A** and **B**). However, for saturating potassium concentrations (above 5 mM), this strain displayed enhanced growth compared to the *hrk1*Δ strain in the presence of acetic acid stress (**Figures 3D** and **E**), indicating the involvement of other Hrk1 targets in acetic acid tolerance.

### Hrk1 mediates the modulation of alkali-metal cation homeostasis

**Figure 4:**
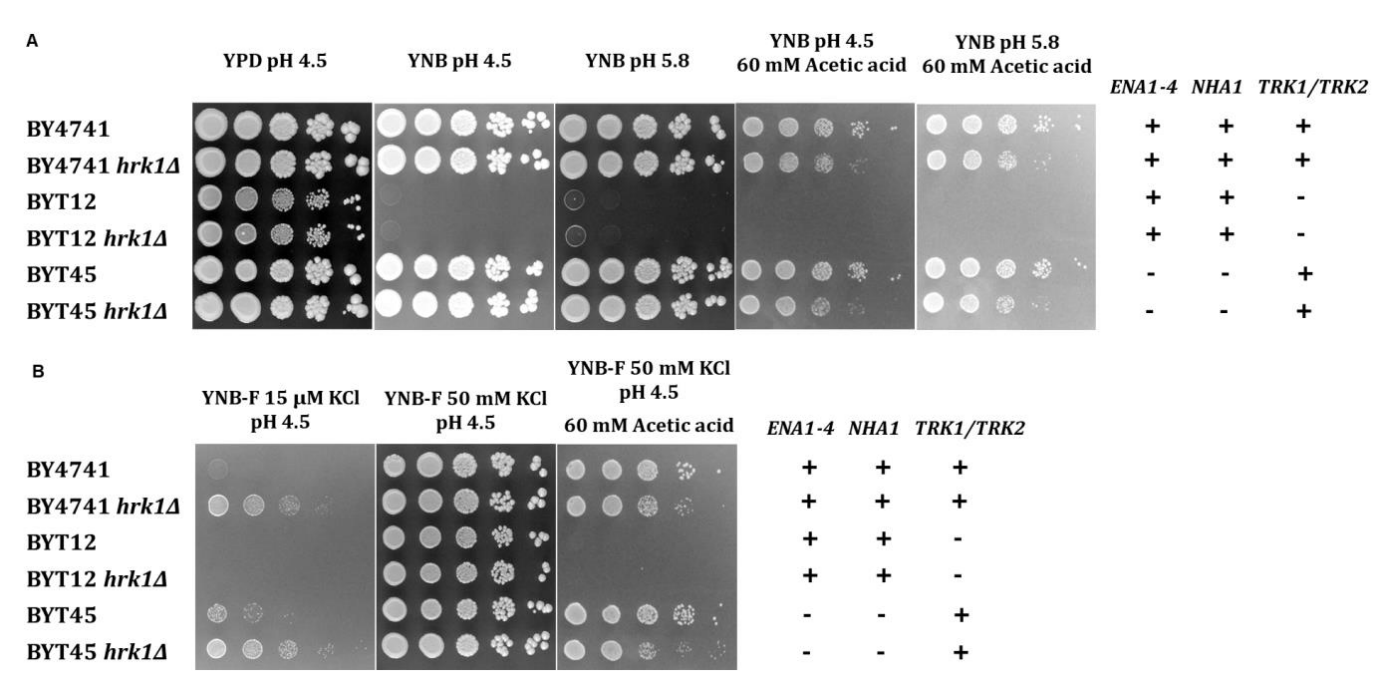
FIGURE 4: Growth phenotypes of *S. cerevisiae* BY4741, BYT12, BYT45, and respective *hrk1*Δ deletion mutants to varying potassium concentrations. Growth of *S. cerevisiae* BY4741 and isogenic mutant strains in media containing varying potassium concentrations (YPD or YNB), in the presence or absence of acetic acid (60 mM at pH 4.5 or 5.8) **(A)**, or in media containing an extremely low potassium concentration (YNB-F, 15 µM), supplemented or not with 50 mM KCl or acetic acid (60 mM at pH 4.5) **(B)**. The dilution series includes strains expressing or not the *HRK1* gene: BY4741 (parental strain), BYT12 (*trk1*Δ *trk2*Δ), BYT45 (*ena1-4*Δ *nha1*Δ). Tenfold serial dilutions (from 10^0^ to 10^−4^) of saturated yeast cultures (OD_600_ 2.0) are displayed.

To assess whether Hrk1 plays a role in influencing potassium homeostasis by modulating potassium uptake or efflux systems, both under acetic acid-stressed and unstressed conditions, experiments were conducted using isogenic strains of the BY4741 background. These strains included deletions of the main potassium efflux systems – the plasma-membrane Ena Na^+^,K^+^-ATPase and the K^+^,Na^+^/H^+^ antiporter Nha1 (BYT45 strain [41]) – or deletions of the main potassium importers – Trk1 and Trk2 (BYT12 strain [60]). In these strains, the *HRK1* gene was additionally disrupted. To evaluate whether the observed effects were directly attributable to acetic acid or influenced by variations in medium pH and composition, cultures of these cells were grown in YPD (rich medium) or YNB (minimal medium) at two different pH levels: 4.5 and 5.8. A concentration of acetic acid of 60 mM for growth in agar plates was used to ensure a level of acetic acid-induced inhibition equivalent to that used in liquid media cultivations. While the potassium concentration in YPD was sufficient to sustain the growth of BYT12 and BYT12 *hrk1*Δ strains, their growth was compromised in YNB medium (containing approximately 15 mM potassium [61]; **Figure 4A**). Notably, the deletion of *HRK1* in BYT12 did not result in any significant alteration in terms of growth enhancement or inhibition, irrespective of whether YPD or YNB media were used. On the other hand, the deletion of the K^+^ efflux transporters (BYT45) did not cause any observable growth hindrance in the presence of acetic acid stress when compared to BY4741. However, deletion of *HRK1* in BYT45, similarly to the effect observed in BY4741, resulted in a similar growth inhibition in both strains, in contrast to their respective parental strain in YNB at pH 4.5 or pH 5.8 supplemented with acetic acid (**Figure 4A**). Under conditions of potassium limitation for both BY4741 and BYT45 strains (using YNB-F medium containing only 15 µM K^+^), the deletion of *HRK1* led to significantly improved growth for both strains (**Figure 4B**). These results are consistent with those shown in **Figure 3A**, comparing BY4741 cultures, expressing or not *HRK1*, cultivated in media with limiting potassium concentrations (1 mM KCl).

**Figure 5:**
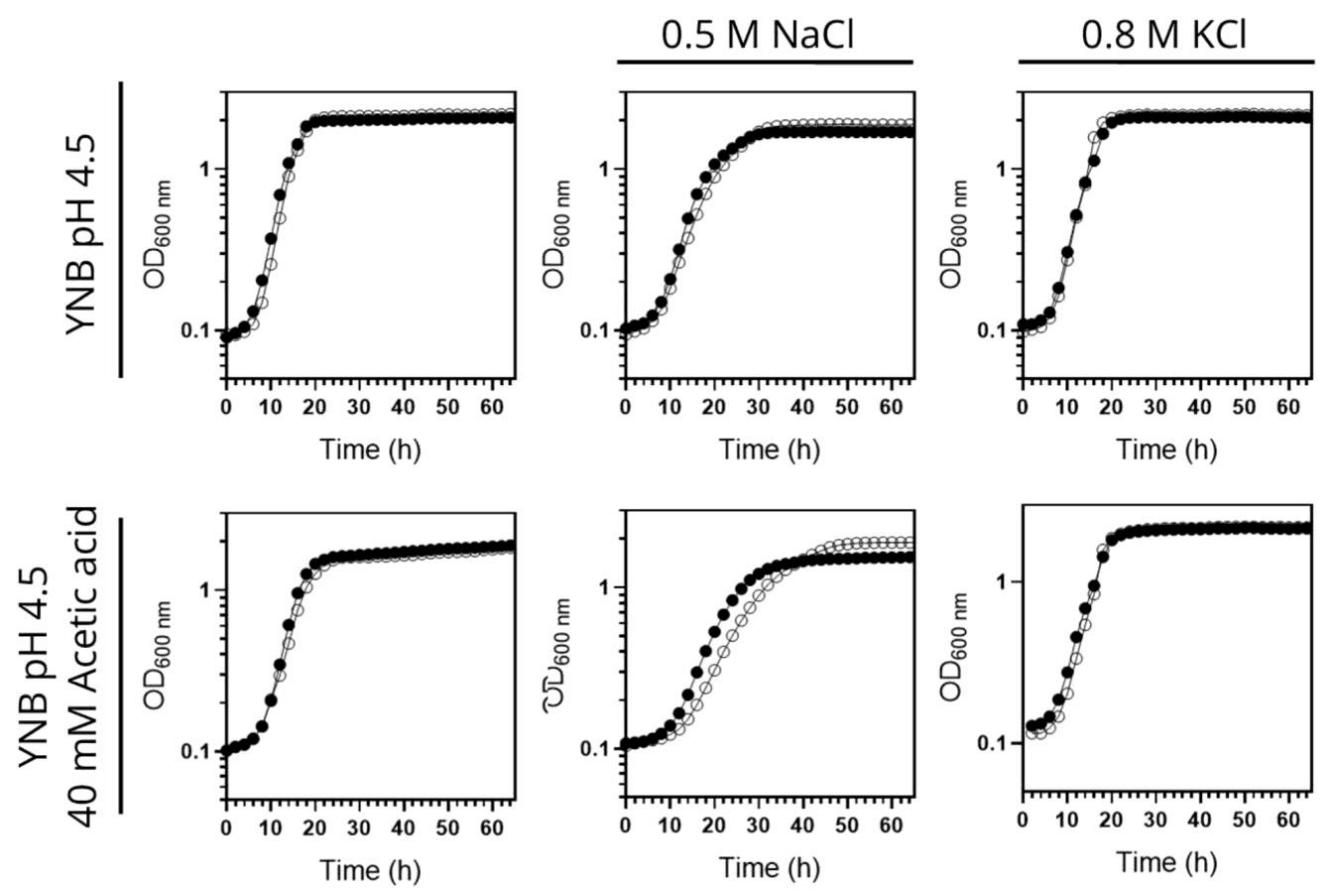
FIGURE 5: Tolerance of *S. cerevisiae* BY4741 and *hrk1*Δ mutant strains to high salt (KCl or NaCl) concentrations in the absence or presence of acetic acid stress. Growth curves of the parental strain BY4741 (●) and the *hrk1*Δ deletion mutant (○), cultivated in YNB pH 4.5 media, supplemented with 0.8 M KCl or 0.5 M NaCl in the presence or absence of acetic acid stress (40 mM at pH 4.5).

**Figure 6:**
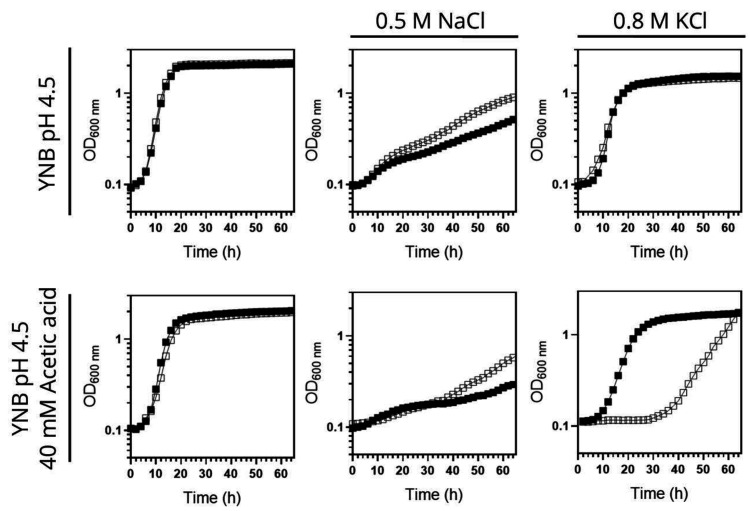
FIGURE 6: Tolerance of *S. cerevisiae* BYT45 and *hrk1*Δ mutant strains to high salt (KCl or NaCl) concentrations in the absence or presence of acetic acid stress. Growth curves of the parental strain BYT45 (■) and *hrk1*Δ deletion mutant (□), cultivated in YNB pH 4.5 media, supplemented with 0.8 M KCl or 0.5 M NaCl in the absence or presence of acetic acid stress (40 mM at pH 4.5)).

To further investigate the role of Hrk1 in maintaining alkali-metal-cation homeostasis, a comparison was made between the salt-sensitive BYT45 strain, expressing or not the *HRK1* gene, and the parental BY4741 strain. Salt (NaCl or KCl) and acetic acid stress tolerances were assessed for both the BYT45 and BYT45 *hrk1*Δ strains, and these were compared with the growth behaviors of the BY4741 and BY4741 *hrk1*Δ strains (**Figures 5** and **6**). The BYT45 strain is known for its sensitivity to elevated salt concentrations, specifically above 1 M KCl and 0.5 M NaCl [62]. Therefore, to ensure the tested conditions allowed the distinction of phenotypic effects in the presence of salts and acetic acid stress, milder concentrations of KCl, NaCl and acetic acid were used. Under the tested salt (0.5 M NaCl and 0.8 M KCl) and acetic acid (40 mM at pH 4.5) concentrations, no significant growth inhibition was observed for BY4741 and BY4741 *hrk1*Δ strains (5). The sole exception was in the presence of 0.5 M NaCl and acetic acid stress, where the BY4741 *hrk1*Δ strain displayed a slight growth inhibition and higher final biomass concentration (**Figure 5**). In contrast, the BYT45 strain displayed distinctive growth patterns. Considering the salt stress condition in the presence of 0.5 M NaCl, deletion of the *HRK1* gene in the BYT45 background led to a significant improvement in growth. This result suggests that the influence of *HRK1* expression on the tolerance to NaCl in the BYT45 strain could be independent of the involvement of sodium efflux systems (**Figure 6**). Furthermore, under salt stress conditions with 0.8 M KCl, a modest growth inhibition in BYT45 was observed compared to BY4741. Deletion of *HRK1* in the BYT45 strain did not significantly alter growth under this condition. However, when salt stress was combined with acetic acid exposure, distinct outcomes were observed. In the presence of 0.5 M NaCl and acetic acid, the BYT45 *hrk1*Δ strain grew better than BYT45. Conversely, the combination of KCl salt stress and acetic acid resulted in an extremely heightened sensitivity of the BYT45 *hrk1*Δ strain, characterized by an extended latency phase during growth in this condition (**Figure 6**). These results underscore the intricate involvement of Hrk1 in the adaptation to acetic acid stress and potassium homeostasis, particularly in conditions where cells lack the main potassium efflux systems.

### Hrk1 mediates the regulation of intracellular pH

**Figure 7:**
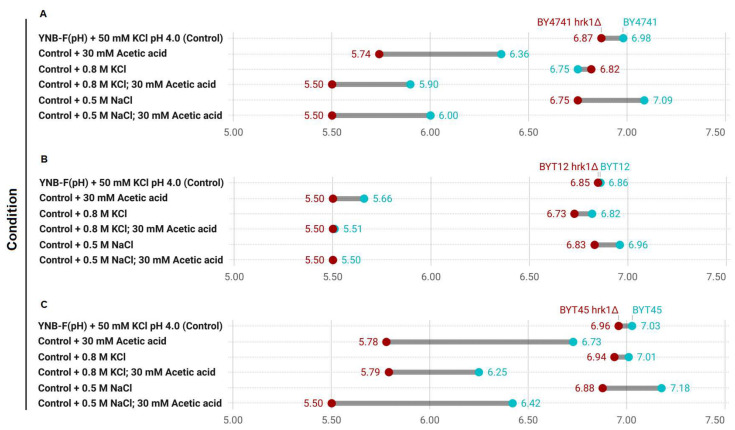
FIGURE 7: Comparison of intracellular pHi estimations in the transient response to salt and acetic acid stresses of *hrk1*Δ deletion mutant strains and the respective parental strains Range plots illustrating the estimated pHi values for each of the tested conditions – 30 minutes exposure to high salt (0.8 M KCl or 0.5 M NaCl) and acetic acid stress (30 mM at pH 4.0) – at each end of the range for the *hrk1*Δ deletion mutant strains (depicted in red), and the respective parental strains (depicted in blue).

The role of Hrk1 in influencing proton and potassium homeostasis was further explored through the assessment of pHi in BY4741 and in strains deleted for the main potassium efflux systems (BYT45; **Figure 7**). For a comprehensive comparison, the BYT12 strain, which lacks the potassium importers Trk1 and Trk2 was also included. The pHi determination was performed under equivalent stress conditions to the ones employed for the growth assays shown in **Figures 5** and **6**. Given the high sensitivity of BYT12 strains to acetic acid stress and the sensitivity range of pHluorin, a 30 mM acetic acid concentration at pH 4.0 was used. The transient alterations of pHi following 30 minutes of exposure to the tested stress conditions were assessed. In the absence of stress, the deletion of *HRK1* yielded a minor decrease in pHi across the three sets of strains. The absence of the potassium import systems (BYT12), caused a reduction in pHi both without stress and during NaCl stress exposure (**Figure 7A** and **B**). Conversely, the deletion of the main potassium efflux systems (BYT45) caused a slight increase in pHi in the absence of stress and during salt stress (KCl or NaCl; **Figure 7A** and **C**), which was aligned with previous studies. [43, 63]. The abrogation of *HRK1* expression triggered significant reductions in pHi within all three strains sets under NaCl stress. Acetic acid exposure, however, led to more pronounced fluctuations in pHi for all strains. In BY4741, exposure to acetic acid resulted in a substantial decline in pHi, a trend further exacerbated by deletion of *HRK1* gene across all tested conditions (**Figure 7A**). BYT12 strains were extremely sensitive to acetic acid, evidenced by the profound intracellular pH decrease, which was even more pronounced in BYT12 *hrk1*Δ (**Figure 7B**). The BYT45 strain, compared to BY4741, exhibited a less pronounced decrease in pHi under acetic acid exposure. Nevertheless, deletion of *HRK1*, also resulted in further reductions in pHi, which were comparatively milder than those observed in BY4741 *hrk1*Δ, especially in the presence of high KCl concentrations (**Figure 7C**). The milder reduction in pHi in the BYT45 strain under acetic acid exposure, as compared to BY4741, suggests that the absence of the main potassium efflux systems might contribute to a partial mitigation of the pH decrease, possibly through increased potassium accumulation and decreased Nha1-dependent influx of H^+^. However, the additional reduction in pHi upon *HRK1* deletion in the BYT45 background implies that it might play a role in fine-tuning pH regulation, potentially through modulation of ion transporters other than the main potassium efflux systems.

## DISCUSSION

**Figure 8:**
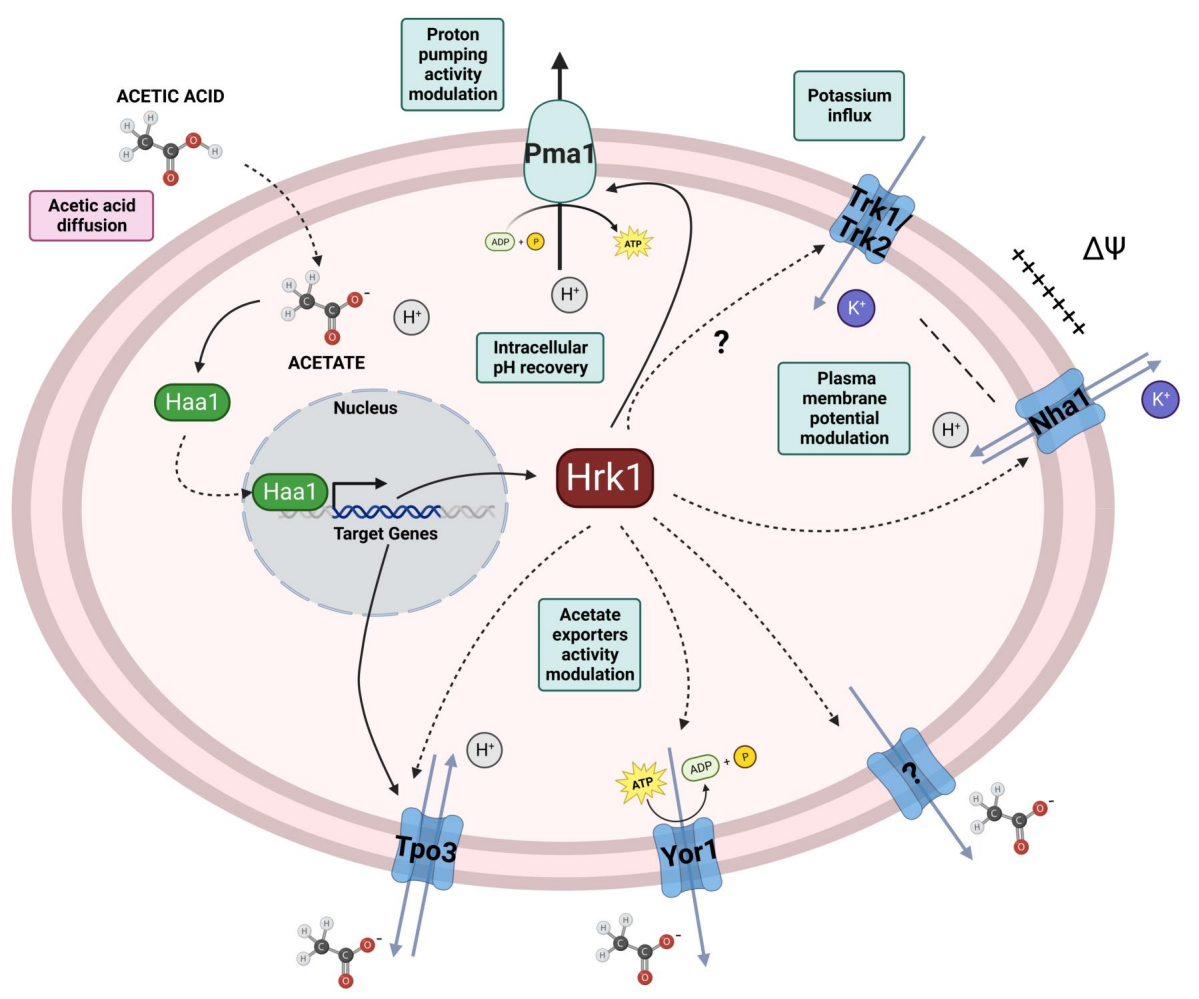
FIGURE 8: Schematic model proposed for Hrk1 involvement in *S. cerevisiae* adaptation and tolerance to acetic acid stress. The model depicts the proposed mechanisms of *S. cerevisiae* adaptation to acetic acid-induced stress that are modulated by Hrk1 based on the results presented here complemented by results from previously published studies. This model focuses on the ion fluxes across the plasma membrane and does not take into account their intracellular distribution.

Understanding the molecular mechanisms underlying acetic acid stress tolerance is essential to guide the improvement of yeast performance and productivity in industrial applications. Notably, Hrk1, belonging to a family of kinases primarily implicated in the regulation of the activity of plasma membrane proteins, has emerged as a key player in yeast's acetic acid stress response, whose underlying mechanisms remain unexplored [47, 64, 65]. This study dives deeper into the intricate relationship between Hrk1 and Pma1 in acetic acid-challenged cells and the role of Hrk1 in H^+^ and K^+^ homeostasis in yeast. Taking into account the results from this study, a schematic model representing the possible mechanisms underlying Hrk1 role in the adaptive response and tolerance of *S. cerevisiae* to acetic acid stress is proposed (**Figure 8**).

Our study involved a time-course analysis of the coordinated activity of the plasma membrane proton pump and pHi during acetic acid-stressed cultivation providing a more comprehensive view of the effect of Hrk1 kinase activity on these responses. This approach allowed the identification of their gradual changes across different growth phases. Results revealed that the activation of plasma membrane H^+^-ATPase activity in yeast cells growing under acetic acid stress was significantly reduced upon deletion of the *HRK1* gene and support the concept that *HRK1* expression plays a role in maintaining pH homeostasis under acetic acid stress. The comparatively less efficient recovery of pHi to more physiological values observed herein in the *hrk1*Δ mutant strain under acetic acid stress aligns with the concurrently registered reduced proton pumping activity of Pma1. The observed impact of *HRK1* gene deletion on Pma1 activity in response to acetic acid suggests an intricate role of Hrk1 in the modulation of H^+^ homeostasis during stressful conditions, enlightening the mechanisms underlying the role of Hrk1 as an important determinant of acetic acid tolerance. This model is consistent with the described increase in *HRK1* expression under acetic acid stress [13], and the role for Pma1 as a major regulator of cytosolic pH [51]. In fact, mutations in the *PMA1* gene leading to diminished proton efflux activity result in growth impairment under acetic acid exposure [66]. The involvement of Hrk1 in the positive regulation of Pma1 activity during the early response to glucose metabolism was previously reported, albeit the extent of the observed activation was modest [21, 67] and not detected in our study performed during cultivation in glucose-repleted medium.

Evidence obtained during this study also indicates the involvement of Hrk1 in potassium homeostasis, which constitutes an important mechanism underlying tolerance to weak acid stress [68], playing a critical role in maintaining intracellular pH homeostasis. To counteract the intracellular acidification caused by the diffusion of weak acids across plasma membrane, yeast cells take up potassium from the extracellular environment, which can be exchanged by protons through K^+^/H^+^-antiporters [46, 47, 68]. Consistent with the importance of K^+^ homeostasis in weak acid tolerance, K^+^ supplementation of a growth medium with vestigial potassium concentrations (YNB-F), was shown here to improve, in a concentration dependent manner, the growth of the parental, and *hrk1*Δ and *nha1*Δ deletion mutant strains under acetic acid stress. Furthermore, when a growth medium with limiting potassium concentrations (1 mM or 2 mM KCl) was used, the *hrk1*Δ strain exhibited a shorter lag phase compared to the parental and *nha1*Δ strains during cultivation under acetic acid stress of these non-adapted yeast cells. The higher tolerance exhibited by the *hrk1*Δ strain comparatively with both the parental and *nha1*Δ strains under conditions of K^+^ limitation in the presence or absence of acetic acid stress suggests a potential link between Hrk1 and the intracellular accumulation of potassium under both conditions. This hypothesis arises from the notion that Hrk1 may exert an influence on Nha1 activity under conditions of K^+^ limitation and acetic acid stress. Nevertheless, it is noteworthy that this hypothesis encounters an intriguing inconsistency when considering the sensitivity phenotype observed in the *nha1*Δ strain during acetic acid stress. Deletion of *NHA1* would presumably be anticipated to diminish the influx of H^+^ and the efflux of K^+^, both of which are perceived as advantageous for adapting to acetic acid-induced stress. However, when considering the yeast cell as a whole, the significance of Nha1 becomes evident in the influence of both the establishment and maintenance of plasma membrane potential. This role, combined with the biological role of specific multidrug resistance efflux transport systems, which is discussed in greater detail below, could be a possible explanation for the observed acetic acid stress-sensitivity of *nha1*Δ. Moreover, the heightened sensitivity phenotype of BYT45 *hrk1*Δ to acetic acid stress in the presence of high KCl concentrations also indicates that Hrk1 does have additional targets beyond Nha1, which are significant in the response to acetic acid-induced stress (**Figure 8**). Another key player contributing to acetic acid tolerance is the potassium importer, Trk1. Its deletion leads to heightened sensitivity to stress induced by acetic acid, whereas overexpression enhances tolerance [45, 47, 68], aligning with the advantageous effects of potassium supplementation on acetic acid tolerance. Nevertheless, the putative influence of Hrk1 in Trk1 activity under acetic acid stress conditions is still unclear.

The observed plasma membrane hyperpolarization in cells lacking *HRK1* under acetic acid-stressed cultivation was also intriguing. This hyperpolarization cannot be attributed to the decreased proton pumping activity in these cells. In fact, a reduction of H^+^-ATPase activity leads to an increased intracellular concentration of protons and a shift in electrochemical equilibrium towards a positive charge increase within the cell, which would be expected to drive plasma membrane depolarization. However, if a systems level perspective is considered, it is known that under acetic acid stress conditions the influx of K^+^ is insufficient to adequately offset the imbalance induced by increased proton pumping activity [68]. Consequently, it has been postulated that anion efflux pathways could potentially contribute to the rectification of this imbalance [68]. Remarkably, the intracellular accumulation of radiolabeled acetic acid in *hrk1*Δ cells was previously reported, revealing a substantial intracellular accumulation of acetate within these cells compared to the parental strain [13]. This high intracellular accumulation of acetic acid counter-ion could potentially contribute to the registered hyperpolarization of the plasma membrane of *hrk1*Δ cells during cultivation under acetic acid stress through an imbalance in charge equilibrium mechanisms. The intracellular acetate accumulation in *hrk1*Δ cells might be a consequence of the disruption of cell surface remodeling mechanisms [69, 70], or the reduction of the activity of acetate exporters. Possible candidates for the active extrusion of acetate, whose activity could be negatively influenced by *HRK1* deletion, are the multidrug transporters Tpo3 and Yor1 (**Figure 8**), identified as potential Hrk1 phosphorylation targets [16]. Tpo3 is an acetic acid-induced plasma membrane drug/H^+^ antiporter involved in acetic acid tolerance by mediating the active efflux of acetate [12, 13]. Interestingly, like *HRK1, TPO3* is part of the Haa1-regulon activated under acetic acid stress. Yor1 is a plasma membrane ABC multidrug exporter with a broad substrate range, including acetate [71, 72].

Notably, *HRK1* deletion was shown here for the first time to have a positive impact on growth under conditions of limited potassium availability. This result suggests a plasma membrane hyperpolarization caused by *HRK1* deletion under conditions of limiting potassium availability since Trk1, the primary potassium importer and responsible for intracellular potassium accumulation, functions as a uniporter driven by the membrane potential [38]. Therefore, growth under potassium-limiting conditions displays a direct correlation with the level of membrane potential [38, 73]. The growth improvement observed when BY4741 *hrk1*Δ is grown under potassium-limiting conditions was also evident in the BYT45 *hrk1*Δ strain, which lacks the potassium exporters Ena and Nha1, respectively. Under conditions of sufficient potassium (above 1 mM), its intracellular concentration is kept at approximately 300 mM, whereas under limiting potassium concentrations (below 1 mM), internal levels correlate with external concentrations [74]. The results from the present study therefore suggest an increase in intracellular potassium accumulation or an enhanced potassium uptake capacity in cells lacking the *HRK1* gene. The absence of growth improvement under limiting potassium concentrations in the BYT12 *hrk1*Δ strain compared to the BYT12 strain, leads to the hypothesis that the observed growth improvement in BY4741 *hrk1*Δ and BYT45 *hrk1*Δ may be dependent on the activity of the Trk1 and Trk2 potassium import systems. This suggests a potential regulatory effect of Hrk1 on Trk1/2 activities. Additionally, Hrk1 might also play a key role in the maintenance of potassium intracellular concentration through the activation or stabilization of the Nha1 antiporter, which is a phosphorylation target of Hrk1 [17]. However, at this stage, we cannot exclude the possibility that the improved growth of *hrk1*Δ under K^+^-limiting conditions is the result of enhanced tolerance to ammonium toxicity. This hypothesis derives from previous findings that suggest ammonium is associated with toxicity in *S. cerevisiae* when subjected to potassium limitation [75]. Moreover, ammonium is reported to impair the growth of *PMA1* mutant strains with decreased proton pump activity, an effect that can be suppressed by KCl supplementation [66]. This intricate scenario might intersect with the function of the multidrug transporter Qdr2, another potential Hrk1 phosphorylation target [16]. Qdr2 is known to play a role in potassium transport and functions independently of the Trk1 and Trk2 transport systems [20].

The interplay between Hrk1, Pma1, and Nha1 biological functions examined in this study suggests a complex network of regulatory mechanisms in response to acetic acid stress. Hrk1 might act as a key mediator, fine-tuning the activity of Pma1 and Nha1 to modulate H^+^ and K^+^ fluxes and, as a result, maintain proper cytosolic pH homeostasis and intracellular potassium concentration levels under acetic acid stress conditions. Further studies into the direct interactions and signaling pathways involving these proteins and exploration of other putative Hrk1 phosphorylation targets, in particular the efflux pumps referred above, will further enlighten the complex role of the Hrk1 kinase in the response to acetic acid stress at a systems level.

## MATERIALS AND METHODS

### Yeast strains and growth conditions

The yeast strains used in this study are listed in **Table 1**. The *HRK1* gene was deleted in *Saccharomyces cerevisiae* BY4741, BYT12, or BYT45 strains by homologous recombination with the Cre-loxP system and the KanMX marker gene [76]. The oligonucleotides used are listed in the Supplementary Table S1. The correct deletion of the *HRK1* gene was verified by PCR using the oligonucleotides listed in the Supplementary Table S1. Strains with an integrated DNA fragment encoding pHluorin (**Table 1**) were constructed as previously described [77] and used for cytosolic pH measurements. The correct integration of the loxP-kanMX-loxPGPD1^*P*^-pHl cassette was verified by PCR.

**Table 1.** *Saccharomyces cerevisiae* strains used in this study.

**Strain**	**Genotype**	**Reference**
BY4741	*MATα his3*Δ*1 leu2*Δ*0 met15*Δ*0 ura3*Δ*0*	EUROSCARF
BYT12	BY4741 *trk1*Δ*::loxP trk2*Δ*::loxP*	[60]
BYT45	BY4741 *nha1*Δ *::loxP ena1-5*Δ *::loxP*	[41]
BY4741 *hrk1*Δ	BY4741 *hrk1*Δ*::loxP*	This study
BYT12 *hrk1*Δ	BYT12 *hrk1*Δ*::loxP*	This study
BYT45 *hrk1*Δ	BYT45 *hrk1*Δ*::loxP*	This study
BY4741 *nha1*Δ	BY4741 *nha1*Δ*::kanMX*	EUROSCARF
BY4741^pHi^	BY4741 *his3*Δ*1::loxP-kanMX-loxP-pGPD1-pHluorin*	[77]
BYT12^pHi^	BYT12 *his3*Δ*1::loxP-kanMX-loxP-pGPD1-pHluorin*	[77]
BYT45^pHi^	BYT45 *his3*Δ*1::loxP-kanMX-loxP-pGPD1-pHluorin*	[63]
BY4741 *hrk1*Δ^pHi^	BY4741 *hrk1*Δ *his3*Δ*1::loxP-kanMX-loxP-pGPD1-pHluorin*	This study
BYT12 *hrk1*Δ^pHi^	BYT12 *hrk1*Δ *his3*Δ*1::loxP-kanMX-loxP-pGPD1-pHluorin*	This study
BYT45 *hrk1*Δ^pHi^	BYT45 *hrk1*Δ *his3*Δ*1::loxP-kanMX-loxP-pGPD1-pHluorin*	This study

All strains were kept at -80°C in appropriate media supplemented with 30% (v/v) glycerol. Prior to use, strains were transferred onto YPD agar plates (2% glucose (Merck), 1% yeast extract (Difco), 2% peptone (Difco), 2% agar) and grown for 24 h at 30°C. Yeast cultures were routinely batch-cultured at 30°C, with orbital agitation (250 rpm), in standard media YPD or YNB (2% glucose (Merck), 0.17% YNB without amino acids and ammonium sulphate (Difco), 0.4% ammonium sulfate (Merck), supplemented with the amino acid mixture OMM (127.22 mg/L L-glutamic acid (Sigma), 47.28 mg/L L-histidine (Sigma), 110 mg/L leucine (Sigma), 149.92 mg/L L-lysine (Sigma), 40 mg/L methionine (Sigma), 50 mg/L phenylalanine (Sigma), 375 mg/L serine (Sigma), 200 mg/L threonine (Sigma) and 40 mg/L uracil (Sigma)) from a 50x concentrated sterile stock solution prepared according to [78] before media sterilization). For the selection of *S. cerevisiae* transformants with the loxP-kanMX-loxP or loxP-kanMX-loxP–GPD1P-pHl cassettes, YPD was supplemented with Geneticin (G418) (1000 µg/mL). To assess growth under limiting or increasing K^+^ con-centrations, a K^+^-free medium, YNB-F (2% glucose (Merck), 0.17% YNB without amino acids, ammonium sulphate and potassium (containing only 15µM K^+^); Formedium), 0.4% ammonium sulphate (Merck), supplemented with the amino acid mixture OMM), was used after supplementation with the indicated KCl concentrations. For intracellular pH measurements, filter-sterilized YNB^pH^ or YNB-F^pH^ medium (2% glucose (Merck), 0.17% YNB without amino acids, ammonium sulphate, riboflavin, folic acid, and potassium (for YNB-F^pH^); Formedium), 0.4% ammonium sulphate (Merck), supplemented with the amino acid mixture OMM) were used after supplementation, when required, with the indicated KCl concentrations. The media pH was adjusted with HCl. For the spot assays, media agar plates were prepared by adding 2% agar.

### Growth assays

Yeast growth was monitored on agar media or in liquid media. For the spot assay on agar media plates, cells pre-grown on YPD or YNB agar plates were resuspended in sterile water to OD_600_ = 0.6 (Spekol 211, Carl Zeiss). Serial 10-fold dilutions of these cell suspensions were prepared and spotted on YPD, YNB or YNB-F agar plates without or supplemented with increasing concentrations of alkali-metal-cation salts. Growth was recorded for up to six days. The results shown are representative results obtained from at least three independent experiments. For the majority of growth experiments in liquid medium, cells were cultivated at 30°C, with orbital shaking (250 rpm) in YNB or YNB-F pH 4.0 supplemented with different KCl concentrations. Growth was followed by measuring the culture optical density at 600 nm (OD_600_). For growth curve measurements under high salt concentrations, cells were inoculated to OD_600_ = 0.05 in 150 µL aliquots of media supplemented or not with 0.8 M KCl or 0.5 M NaCl in the presence and absence of acetic acid (40 mM at pH 4.5) in a 96-well microplate and cell growth was monitored in a SPECTROstar^*Nano *^ Microplate Reader (BMG Labtech). Two technical replicates were registered for each strain and set of conditions within individual experiments. The results are shown as a mean of two independent experiments.

### Plasma membrane H^+^-ATPase activity

#### Cell Collection and Total Membrane Fraction Preparation

*S. cerevisiae* BY4741 and BY4741 *hrk1*Δ cells were collected at selected times during growth, in the absence or presence of acetic acid (50 mM, pH 4.0), using rapid filtration. The pellets were resuspended in their supernatants to a standardized cell density of 40 units of OD_600_. TRIS, EDTA, and dithiothreitol were added to final concentrations of 100 mM, 5 mM, and 2 mM, respectively [29]. The cell suspensions were then rapidly frozen at -80°C and stored until use. After thawing, cell suspensions were vortexed with acid-washed glass beads in eight bursts of 1 min each, with 1 min on ice between bursts. The homogenates were diluted with 5 mL of 0.33 M sucrose, 5 mM EDTA, 2 mM dithiothreitol, and 0.1 M Tris-HCl (pH 8.0) and centrifuged for 3 min at 1000 g. The supernatant was centrifuged for an additional 5 min at 3000 g, and the resulting supernatant was then centrifuged for 40 min at 20,000 g. The pellet of membranes was resuspended in 300 µL of suspension buffer containing 20% (v/v) glycerol, 0.1 mM EDTA, 0.1 mM dithiothreitol, and 10 mM TrisHCl (pH 7.5). The total plasma membranes were stored at -80°C until further use. Protein concentrations were determined using the Pierce^™^ BSA Protein Assay Kit with bovine serum albumin (BSA) as a standard.

#### ATPase assay

Plasma membrane H^+^-ATPase activity was measured as previously described [79], using an Mg-ATP concentration of 2 mM. ATPase activities were calculated by linear regression from the slope of released Pi versus time (0, 5, 10, 15, and 20 min). The results were expressed as nmol Pi released per min (mU) and per mg of total membrane protein. Results are displayed as representative from at least two other independent experiments for each strain and set of conditions leading to similar results.

### Intracellular pH estimations

Yeast strains expressing pHluorin were grown in YNB^pH^ or YNB-F^pH^ + 50 mM KCl with an initial OD_600_ of approximately 0.5. Fluorescence intensities were recorded at selected time points using a FilterMax F5 Multi-Mode Microplate reader (Molecular Devices) with an emission filter of 534 nm and excitation filters of 405 nm and 465 nm. The ratio of emission intensity I405 nm/I465 nm was used to calculate the intracellular pH according to a calibration curve prepared as previously described [80]. Each strain was measured in three wells (200 µL of cells per well) within one experiment (technical replicates), and the presented data are average values ± SD of at least two independent experiments (biological replicates).

### Plasma membrane potential measurements

The plasma membrane potential was estimated by measuring the fluorescence intensity of cells exposed to the fluorescent probe carbocyanine 3,3'-dihexyloxacarbocyanine iodide (DiOC_6_(3)) [81]. Cells were cultivated in YNB media and collected at selected time points along the growth curve. Cell pellets were washed in MES/glucose buffer (10 mM MES, 0.1 mM MgCl_2_, and 20 g/L glucose [pH 5.6]) and resuspended in MES/glucose buffer supplemented with 200 nM DiOC_6_(3) (Molecular Probes). The cells were then incubated in the dark for 15 min at 30°C with orbital agitation (100 rpm). After centrifugation, cells were resuspended in MES/glucose buffer and analyzed using flow cytometry with a BD Accuri^™^ C6 Plus (BD Biosciences) and a FL1 560/20 nm filter. All experiments were repeated using at least two biological replicates, with samples being stained and analyzed in duplicate. A fixed total of 50,000 events per sample were acquired using a slow flow rate (14 µl min^−1^).

## SUPPLEMENTAL MATERIAL

Click here for supplemental data file.

All supplemental data for this article are available online at https://www.microbialcell.com/researcharticles/2023a-antunes-microbial-cell/.

## Abbreviations:

ABC: – ATP-binding cassette,
MDR: – multi drug resistance,
pHi: – intracellular pH.
