# Supplementary material for: The Hrk1 kinase is a determinant of acetic acid tolerance in yeast by modulating H+ and K+ homeostasis
Source: Microb Cell. 2023 Nov 14;10(12):261–76. doi: 10.15698/mic2023.12.809 (PMC10695635; doi:10.15698/mic2023.12.809)
Supplement: Supplementary file 1 [file mic-10-261-s01.pdf]

TABLE S1. Oligonucleotides used in this study.

Oligonucleotide	Sequence
**Deletion of *HRK1***	
Hrk1_F1	5'-CGCTCACCATACTCTTGCATTAG-3'
Hrk1_R1	5'-GAGGATGCTGGGCACGATAGTG-3'
Hrk1_F2	5'-CGAGTCAACAGCACAGAGGACC-3'
Hrk1_R2	5'-CAGTGAGAAATGGGGTGTCAAC-3'
Hrk1-KanMX-F1	5'-CGGTAGAACTATTTCTCGTATAAAGATGCCTAATCTATTGTTCGTACGCTGCAGGTCGAC-3'
Hrk1-KanMX-R1	5'-CTCTATATTGTATGTATAATATGATAGATACTCTTCCGTAGCATAGGCCACTAGTGGATCTG-3'
**pHluorin Integration**	
IntgpHl Fwd	5'-GCAGAAAGCCCTAGTAAAGCGTATTACAAATGAAACCAAGTTCGTACGCTGCAGGTCGAC-3'
IntgpHl Rev	5'-CCATTGGGCGAGGTGGCTTCTCTTATGGCAACCGCAAGAGGAAAGAGTTACTCAAGAATAAG-3'
